# Sexual dysfunction and fertility-related distress in young adults with cancer over 5 years following diagnosis: study protocol of the Fex-Can Cohort study

**DOI:** 10.1186/s12885-020-07175-8

**Published:** 2020-08-05

**Authors:** L. Wettergren, L. Ljungman, C. Micaux Obol, L. E. Eriksson, C. Lampic

**Affiliations:** 1 Department of Women’s and Children’s Health, Karolinska Institutet, SE-171 77 Stockholm, Sweden; 2 Department of Learning, Informatics, Management and Ethics, Karolinska Institutet, SE-171 77 Stockholm, Sweden; 3 School of Health Sciences, City, University of London, London, EC1V 0HB UK; 4 Department of Infectious Diseases, Karolinska University Hospital, SE-141 86 Huddinge, Sweden; 5 Department of Public Health and Caring Sciences, Uppsala University, SE-751 22 Uppsala, Sweden

**Keywords:** Cancer, Cohort study, Fertility-related distress, Sexual function, Young adults

## Abstract

**Background:**

There is a lack of firm knowledge regarding sexual problems and fertility-related distress in young adults following a diagnosis with cancer. Establishing such understanding is essential to identify patients in need of specific support and to develop cancer care accordingly. This study protocol describes the Fex-Can Cohort study, a population-based prospective cohort study investigating sexual dysfunction and fertility-related distress in young adults diagnosed with cancer in Sweden. The primary objective of the study is to determine the prevalence and predictors of sexual dysfunction and fertility-related distress following a cancer diagnosis in young adulthood compared to prevalence rates for the general population. Further aims are to investigate the trajectories of these issues over time, the co-existence between sexual dysfunction and fertility-related distress, and the relation between these issues and body image, anxiety and depression, health-related quality of life, self-efficacy related to sexuality and fertility, and fertility-related knowledge.

**Methods:**

Participants in the Fex-Can Cohort will be identified via the Swedish National Quality Registries for Brain Tumors, Breast Cancer, Gynecological Oncology, Lymphoma, and Testicular Cancer. All patients diagnosed at the ages of 18–39, during a period of 18 months, will be invited to participate. Established instruments will be used to measure sexual function (PROMIS SexFS), fertility-related distress (RCAC), body image (BIS), anxiety and depression (HADS), and health-related quality of life (QLQ-C30); Self-efficacy and fertility-related knowledge will be assessed by study-specific measures. The survey will be administered to participants at baseline (approximately 1.5 year after diagnosis) and at 3 and 5 years post-diagnosis. Registry data will be used to collect clinical variables. A comparison group of 2000 young adults will be drawn from the Swedish population register (SPAR) and subsequently approached with the same measures as the cancer group.

**Discussion:**

The study will determine the prevalence and predictors of sexual dysfunction and fertility-related distress in young men and women with cancer. The findings will form a basis for developing interventions to alleviate sexual problems and fertility-related distress in young adults with cancer in the short and long term.

**Trial registration:**

This is an observational cohort study and clinical trial registration was therefore not obtained.

## Background

Cancer affects large groups of young adults, commonly defined as those between 18 to 39 years of age. Globally about one million young adults are diagnosed with cancer yearly, and the corresponding figure in Sweden is approximately 2000 [1, 2]. In addition to being a life-threatening condition, cancer and its treatments may impair several aspects of the general health, including sexual and reproductive functions. Being diagnosed with cancer during young adulthood can thus be particularly distressing by interfering with important life goals, such as establishing intimate relationships and building a family [3].

Previous research has reported that over 40% of young adults with cancer experience sexual problems within the first 2 years following diagnosis [4, 5]. Problems commonly reported by women include reduced sexual desire, vaginal dryness and/or dyspareunia, difficulties in sexual arousal and/or orgasm, and low satisfaction with sex life [6–8]. In men diagnosed with cancer erectile dysfunction, orgasmic difficulties, reduced sexual interest, and low satisfaction with sex life have been reported [9–12]. Sexual problems can be caused by several of the cancers common in the age group and their treatments (i.e., radiation therapy, chemotherapy, endocrine treatment, and surgery), directly or indirectly via physiological, psychological, and interpersonal factors [13, 14]. However, firm knowledge about the mechanisms involved in sexual problems after cancer in young adulthood is not yet established. Previous research has indicated that female gender, higher age, a poor prognosis, and being in a partner relationship predict more sexual problems [4].

Several cancer types and their treatments may cause temporary or permanent infertility or subfertility [15] but fertility potential on an individual level often remains uncertain following cancer in young adulthood [16, 17]. Results indicate that a majority of young women diagnosed with cancer experience fertility-related distress [7, 18], which has been shown to be related to long-term depressive symptoms [19]. In men fertility-related distress following cancer has been studied to a very limited extent. One recent study found that 28% of young men with testicular cancer reported high levels of reproductive concerns approximately 2 years post-diagnosis [11]. In addition, impaired fertility after testicular cancer appears to be related to decreased quality of life and to lower emotional well-being [20, 21]. In recent research, fear of infertility, or knowing that one’s fertility has been compromised, has been associated with negative effects on psychological wellbeing in men diagnosed with various types of cancer [22]. It has also been reported that the threat of infertility is associated with compromised self-esteem, sexuality and body-image in both men and women diagnosed with cancer in young adulthood [11, 16].

Several cancer diagnoses and/or their treatments have potentially negative consequences on fertile ability or sex life, including diagnoses that are common in young adults: brain tumors, breast cancer, cervical cancer, leukemia, lymphoma, ovarian cancer and testicular cancer [1]. Still, research on reproductive and sexual health issues in this group is limited and there is a lack of longitudinal, large-scale studies using validated instruments and reliable comparison data. As a result, knowledge about the prevalence, predictors and trajectory of sexual problems and fertility distress following a cancer diagnosis in young adults is sparse. High quality longitudinal research is needed to advance knowledge necessary to develop cancer care adapted to the needs of this group.

### The Fex-Can project

The project Fertility and Sexuality following Cancer (Fex-Can) includes a cohort study with an embedded randomized controlled trial (RCT) evaluating the effect of a web-based intervention addressing sexual problems and fertility-related distress, see study protocol for the RCT [23]. This intervention was developed and evaluated regarding its feasibility in collaboration with a group of former cancer patients and significant others [24, 25]. The present protocol describes the procedures for the Fex-Can Cohort.

### Objectives

The primary objective of the present study is to determine the prevalence and predictors of sexual dysfunction and fertility-related distress following a cancer diagnosis in young adulthood compared to prevalence rates for the general population. Further aims are to investigate the trajectories of these issues over time, the co-existence between sexual dysfunction and fertility-related distress, and the relation between these issues and body image, anxiety and depression, health-related quality of life, self-efficacy related to sexuality and fertility, and fertility-related knowledge.

## Methods/design

### Study design

The study will have a population-based prospective cohort design, investigating sexual dysfunction and fertility-related distress in young adults diagnosed with cancer over 5 years following diagnosis. The study will also include a cross-sectional assessment of a comparison group, consisting of young adults from the general population.

### Setting

**Fig. 1:**
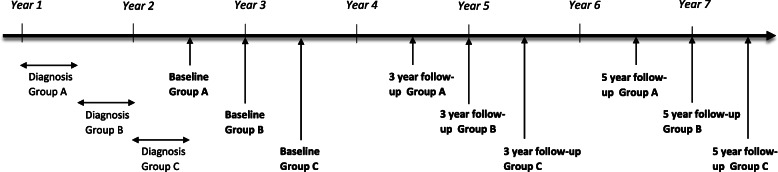
Fex-Can Cohort timeline for data collection of the cancer group

The diagnoses included in the Fex-Can Cohort are selected based on the diseases and/or treatments having potentially negative consequences on fertile ability or sexual life. The incidence of the selected diagnoses in Sweden in 2016 was: brain tumors (*n* = 153), breast cancer (*n* = 350), cervical cancer (*n* = 195), lymphoma (*n* = 132), ovarian cancer (*n* = 39), and testicular cancer (*n* = 220). Individuals diagnosed with leukemia were not included in the Fex-Can Cohort due to an ongoing study concerning fertility issues in this group. Participants will be identified via the Swedish National Quality Registers for Brain Tumors, Breast Cancer, Gynecological Oncology, Lymphoma, and Testicular Cancer. All individuals diagnosed with the selected diagnoses at ages 18–39 during a time period of 18 months will be approached regarding study participation. Data collection will be performed approximately 1.5 years after diagnosis (baseline assessment) and 3 and 5 years after diagnosis. At baseline most participants are expected to have completed first-line treatment and be in the phase of returning to work and studies. In order to time these data assessments to participants’ time of diagnosis, data collections will be performed in three waves (A-C), see Fig. 1. Data for the comparison group will be collected on one occasion.

### Recruitment

#### Cancer group

All individuals matching the inclusion criteria (see below) will be approached regarding study participation with a letter outlining the aims and procedures of the study, the voluntary nature of participation, and a postal survey. The survey will be possible to complete on paper or via the web by using a unique participant code. On request, participants may also have the possibility to report their responses by phone. Two reminders will be sent to non-responders. Participants will be offered two cinema tickets (total value of approximately 20 Euro) as incentives for completion of each assessment (baseline and follow-ups).

#### Comparison group

A random sample of 2000 young adults (1000 women and 1000 men) will be drawn from the Swedish population register (SPAR) and approached regarding study participation. The survey will be sent to potential participants, together with a letter with information about the study including the voluntary nature of participation. As for the cancer group, it will be possible to complete the survey on paper, via the web or telephone, and two reminders will be sent to non-responders. The comparison group will be offered the same incentive for participation as the cancer group i.e., two cinema tickets. The comparison group will only be assessed once.

### Eligibility criteria

#### Cancer group

The following inclusion criteria will be used: All individuals in Sweden in ages 18–39 who were diagnosed with brain tumor, breast cancer, cervical cancer, lymphoma, ovarian cancer, or testicular cancer between January 2016 and August 2017. Potential participants without valid address information will be excluded. Furthermore, approached individuals who on their own initiative inform us that they cannot complete the survey due to cognitive impairment, poor health or non-ability to read and/or understand Swedish will also be excluded.

#### Comparison group

For the comparison group the inclusion criteria will be: Age 19–40 (matching the age of the cancer group at baseline assessment) and registered as residents in Sweden. Furthermore, similar to the cancer group, approached individuals who on their own initiative inform us that they cannot complete the survey due to cognitive impairment, poor health or non-ability to read and/or understand Swedish, will be excluded.

### Variables

The primary outcomes will be sexual function and fertility-related distress. Secondary outcomes will be body image, anxiety and depression, health-related quality of life, self-efficacy related to sexuality and fertility, and fertility-related knowledge. Primary and secondary variables will be collected in the survey. Before conducting the Fex-Can Cohort, the primary outcomes were tested in two pilot studies: one including young women diagnosed with breast cancer (*n* = 181) [7], and one including young men diagnosed with testicular cancer (*n* = 111) [11]. The results showed the instruments to be well accepted. All primary and secondary outcomes will be included in the survey at each assessment. Additionally, background variables (see below) will be collected via the survey at baseline and at follow-ups. Clinical variables (diagnosis, stage, treatment, relapse) will be extracted from registry data and updated in connection with each data collection. See Table 1 for overview of assessments.

**Table 1:** Overview and timing of assessments in the Fex-Can Cohort

Type of data	Time of diagnosis / treatment start	Baseline 1.5 years	Follow-up 3 years	Follow-up 5 years
Mode of administration				
Survey data				
Background variables		X	X	X
Sexual function (SexFS v2)		X	X	X
Fertility-related distress (RCAC)		X	X	X
Anxiety and depression (HADS)		X	X	X
Health-related quality of life (EORTC QLQ-C30)		X	X	X
Body Image (BIS)		X	X	X
Self-efficacy		X	X	X
Fertility-related knowledge		X	X	X
Clinical data	X^a^	X^b^	X^b^	X^b^

^a^Extraction of data from respective quality registry at time of baseline-assessment

^b^Updated information extracted from the quality registries in connection to analyses

#### Cancer group – survey

##### Background variables

Background variables collected in the survey will include sociodemographic information on country of birth, educational level, occupation, partner relationship, children, and sexual orientation. Information on current cancer treatment and the use of fertility preservation procedures will also be included.

##### Sexual function

The Patient-Reported Outcomes Measurement Information System© Sexual Function and Satisfaction Measure version 2 (SexFS v2) is a measure assessing sexual function and satisfaction in men and women regardless of sexual orientation [26]. Items in the SexFS v2 are scored on a five-point scale (ranging from 1 = None/Not at all to 5 = Very/A lot). In this study four specific domains for women will be included: Vaginal lubrication, Vaginal discomfort, Vulvar discomfort – clitoral, and Vulvar discomfort – labial. For males, the specific domain Erectile function till be used. Additionally, four gender-neutral domains will be included for all participants: Interest in sexual activity, Orgasm – ability, Orgasm – pleasure, and Satisfaction with sex life. Item response theory is used to calculate domain scores, which are transformed to a T-score metric where 50 represents the mean for sexually active American adults (standard deviation = 10) [26]. The SexFS v2 has shown adequate content, construct and known-groups validity as well as test-retest reliability [26, 27]. The selected items and domains of the SexFS v2 were translated into Swedish and linguistically validated in accordance with the procedure developed by FACITrans and PROMIS [28].

##### Fertility-related distress

Fertility-related distress will be assessed using the Reproductive Concerns After Cancer (RCAC) scale. The RCAC is a multidimensional measure, assessing a range of concerns related to fertility and parenthood, developed and evaluated for young adult female cancer survivors [29] and recently adapted for male cancer survivors [30]. The scale includes 18 items in six dimensions (3 items each) scored on a five-point scale (ranging from 1 = Strongly disagree to 5 = Strongly agree). The following dimensions are included in the RCAC: Fertility potential, Partner disclosure, Child’s health, Personal health, Acceptance, and Becoming pregnant/Achieving pregnancy. In each dimension, a high level of reproductive concerns reflects fertility-related distress and is defined as a mean score > 4. The RCAC has demonstrated satisfactory internal consistency and construct validity [19, 30, 31]. The original scale for females was translated into Swedish by two bilingual researchers. In parallel to this, a Swedish version for males was developed in collaboration with Dr. Gorman, creator of the original RCAC. Subsequently, these versions were evaluated by one bilingual panel (*n* = 4), one lay panel (*n* = 7) and one patient panel (*n* = 8), as well as by cognitive interviews with 3 young persons with a cancer experience. The Swedish versions have been used in women with breast cancer [7] and men with testicular cancer [11] and shown to be well accepted. Internal consistency in the female version was shown to be good with exception for ‘Becoming Pregnant’ with a Cronbach’s α coefficient of 0.54 [7]. In the male version, internal consistency was acceptable (Cronbach’s α coefficients: 0.64–0.90) in all dimensions [10].

##### Body image

Body image will be assessed with the Body Image Scale (BIS) that measures perception of one’s body image associated with cancer and cancer treatment [32]. The BIS comprises 10 items and responses are given on a four-point scale (ranging from 0 = Not at all to 3 = Very much) with higher scores indicating a more negative body image. Total summary scores can range between 0 and 30, and a total score exceeding 10 is suggested to reflect a negative body image reaching a clinical level [32, 33]. The BIS has shown high test-retest reliability and satisfactory internal consistency in cancer patients [32].

##### Anxiety and depression

The Hospital Anxiety and Depression scale (HADS) measures anxiety and depression in two subscales [34]. Each subscale consists of 7 items and responses are given on a four-point scale (ranging between 0 and 3) with higher scores indicating more distress. Subscale scores can range between 0 and 21, with scores above 7 indicating borderline or clinically significant cases of anxiety or depression, respectively. The subscales have been reported to have satisfactory internal consistency and the concurrent validity has been reported to be good to very good [35].

##### Health-related quality of life

The EORTC QLQ-C30 (version 3.0) will be used to measure health-related quality of life [36, 37]. The instrument includes five functional scales, three symptom scales, a global health status scale, and six single items. All scores will be linearly transformed to a score between 0 and 100. For the functional and the global QoL scales, higher scores indicate better health. For the symptom scales, higher scores indicate more symptom burden. The scale has demonstrated good psychometric properties in cancer populations [36, 38].

##### Self-efficacy

Self-efficacy related to sexuality and fertility will be assessed by study-specific questions measuring confidence in one’s own ability to handle situations, thoughts and emotions related to sexuality (6 items) and to the threat of infertility (6 items). Examples of statements assessing self-efficacy are “I feel confident that I can handle negative thoughts and emotions in relation to my sex life” and “I feel confident that I can cope with meeting friends or relatives who are pregnant”. The items are scored on a four-point scale (ranging from 1 = Strongly disagree to 4 = Strongly agree) and an additional response alternative “Not relevant”. Total mean scores will be calculated, with higher scores indicating higher levels of self-efficacy related to sexuality and fertility, respectively.

##### Fertility-related knowledge

Perceived level of knowledge about general and cancer-related fertility issues will be examined by a study-specific questionnaire with 10 items rated on a four-point scale (ranging from 1 = Disagree completely to 4 = Agree completely). Examples of items are: “I have good knowledge regarding the chance of becoming pregnant at one attempt” and “I have good knowledge regarding the effect of cancer and cancer treatments on reproductive ability”. Total mean scores will be calculated, with higher scores indicating higher levels of perceived fertility-related knowledge.

#### Cancer group - registry data

After receiving formal consent from each registry, the following clinical data will be collected from the Swedish National Quality Registries for Brain Tumors, Breast Cancer, Gynecological Oncology, Lymphoma, and Testicular Cancer: date of diagnosis, clinical stage, type of treatment, relapse, adverse events, secondary cancers and performed fertility preservation. The clinical variables were selected in close collaboration with representatives from each National Quality Registry.

#### Comparison group - survey

The survey administered to the comparison group will include the same instruments as for the cancer group with the exception of the study-specific measures of fertility-related knowledge and self-efficacy related to fertility. Furthermore, specific items related to having had cancer will be deleted from the BIS (5 items) and the RCAC (9 items constituting 3 dimensions: Partner disclosure, Child’s Health, Personal Health). The shortened versions of the BIS and RCAC have not been validated. However, the shortened BIS has been used in a previous study on sexual functioning in the general Dutch population showing a Cronbach’s α coefficient of 0.86 [39].

#### Administration of instruments

Study-specific items and instruments to the cancer group will be administered in the same order at all assessments: BIS; RCAC; Self-efficacy Fertility; Fertility-related knowledge; Self-efficacy Sexuality; SexFS v2; HADS; and EORTC QLQ-30. The comparison group will only be assessed once with study-specific items and instruments, given in the same order as for the cancer group.

### Sample size

Based on official statistics on cancer incidence in Sweden [2, 40] the eligible population is estimated to approximately 1500 for the inclusion period. Based on our experience of moderate response rates (50–60%) in surveys on sensitive issues in this age group when no incentives were offered [7, 11], we expect the addition of incentives (2 cinema tickets) to result in a larger proportion of responders. An estimated response rate of 70% would result in 1050 participants at baseline. At baseline, a majority of participants (≈80%, *n* = 840) are expected to rate sexual dysfunction or fertility-distress meeting the inclusion criteria for the embedded RCT [23]. Of those invited to the RCT, about half are expected to consent participation (*N* = 420) and these will be excluded for further follow-up in the Fex-Can Cohort, leaving 630 participants for the longitudinal analyses. Attrition in the Fex-Can Cohort due to deaths and other reasons for non-response is estimated to 15% at following assessments, giving an estimated response rate at T2 (*n* = 535) and T3 (*n* = 454). Sample size determination was based on the recommendation to include at least 5 events of the dependent variable of interest (in this case: the primary outcome measures SexFS and RCAC) for each independent variable included in the multivariable logistic regression models [41]. Thus, we estimated that at least 50 events of the dependent variable in the sample are required in order to include up to ten independent variables. Based on the incidence rates of the selected diagnoses, we expected a distribution of approximately 65% women and 35% men in the eligible sample. Previous data of sexual dysfunction and fertility distress in the Swedish setting [7, 11] indicate that the number of events of the dependent variables at baseline is ≈30% for males and ≈60% for females. Based on these numbers, we estimate the sample size to be sufficient for determination of potential predictors for both sexes at all assessment occasions, including the 5-year follow-up. As the Fex-Can Cohort is an observational study, no formal power calculation was conducted.

### Statistical methods

The study will be reported following the STROBE statement [42] and the SPIRIT-PRO Extension [43]. Descriptive statistics will be used to determine the prevalence of sexual dysfunction and fertility-related distress by diagnosis and sex. These will be presented as means and standard deviations and as percentages of participants above the described cut-offs for these outcomes. Prevalence rates in the cancer group will be compared to prevalence rates in the general population by sex and age group, using Students’ t-test and χ^2^ tests. To determine predictors for sexual dysfunction and fertility-related distress at each assessment we will perform logistic regression models for each primary outcome (SexFS v2-domains and RCAC-dimensions) for the whole group and by sex. Independent variables will include sex (whole group), partner status, parenthood status, child wish (only for RCAC), satisfaction with sex life pre-diagnosis (only for SexFS v2), diagnosis, treatment intensity, body image, anxiety, and depression. Trajectories of these issues over time (T1, T2 and T3) will be analyzed with linear mixed models. Relations between sexual dysfunction and fertility-related distress, and between these issues and our secondary outcomes, will be analyzed with Pearson’s correlation coefficients. Statistical analyses will be performed in collaboration with external statisticians.

### Ethics and dissemination

#### Research ethics approval

Ethical approval has been obtained for the study procedures by the Regional Ethical Review Board in Stockholm, Sweden (Dnr: 2013/1746–31/4; 2014/2244–32; 2017/916–32; 2017/1416–32).

#### Confidentiality

All participants will receive a unique code number indicated on the survey. The code key will be stored separate from the research data and will only be accessible by members of the research team. All data will be handled and stored according to the EU General Data Protection Regulation (GDPR). This includes storage of paper records in locked spaces on institution premises and storage of electronic records on secure, password-protected servers, with access restricted to the research team. Data will be shared with external statisticians through secure servers. The research team members have formal training in research ethics, which is a mandatory part of doctoral education at the institution. Adherence to research ethics and the study protocol will be monitored by the principal investigators (first and last authors) at regular project meetings and in their supervision of doctoral students and post-doctoral researchers involved in the Fex-Can project.

#### Dissemination policy

The results from the study will be communicated to the scientific, clinical and patient communities through open-access publications in scientific peer-reviewed journals. Additionally, presentations of the results will be made at international and national clinical and scientific conferences and in other contexts.

## Discussion

This population-based cohort study aims to determine the prevalence and predictors of sexual dysfunction and fertility-related distress in young adults diagnosed with cancer. The results of the study will increase understanding of the trajectories of sexual dysfunction and fertility-related distress over 5 years following diagnosis.

Our study design includes a large nationwide sample of young adults diagnosed with different cancers and will therefore establish prevalence rates of sexual dysfunction and fertility-related distress over the first 5 years after diagnosis. One of the selected diagnoses, brain tumors, is a cancer often excluded in this kind of research and the group’s sexual dysfunction and fertility-related distress is still largely unknown. Potential participants will be identified through National Quality Registers with excellent coverage, ensuring that all individuals in the age group diagnosed with the selected cancers will be approached [44]. The design also allows for analyses of non-responders and attrition including highly reliable clinical variables. At the baseline assessment some patients may still be on treatment (e.g. lymphoma and breast cancers) and others will have finished their treatment (e.g. testicular cancer). Therefore, treatment status will be described in detail for each diagnosis when reporting prevalence at baseline, and all models will control for current treatment status. While most of the selected patient-reported outcome measures are standardized instruments, it should be noted that the study-specific measures, as well as the shortened versions of the BIS and RCAC for the comparison group, have not been validated. With a young adult comparison group assessed with the same standardized measures it will be possible to determine to what extent the self-rated problems are related to being treated for cancer. As the prevalence of sexual dysfunction in women and men has been reported to vary between countries [45, 46], the use of a comparison group randomized from the total general population in the country is a strength. However, the fact that the comparison group is only assessed at one time point is a limitation as it does not allow comparison of trajectories of these issues over time.

There are also a few challenges to be considered. Achieving high response rates to surveys targeting cancer populations and the general population have become challenging particularly among young people. In addition, attrition may introduce bias and limit the possibility to perform subgroup analyses. We have tried to minimize this risk by offering the choice of answering the survey on paper, the web or via a telephone interview and offer incentives for each answered survey to reach the highest possible response rate. Furthermore, great care has been taken to phrase written information in order to optimize inclusion of both men and women in the study, as well as individuals with different levels of education. The survey is only available in Swedish, and those who do not understand Swedish (cancer group and comparison group) will be not be able to participate in the study. However, we do offer the possibility to answer the questions by phone to facilitate participation for those who understand Swedish but do not read the language.

To conclude, the Fex-Can Cohort study will elucidate concerns and problems related to sexual life and fertility, as experienced by young adults with cancer. The results will inform different groups of stakeholders including healthcare providers, patients and their partners. The findings will form a basis for developing interventions to alleviate sexual problems and fertility-related distress in young adults with cancer in the short and long term.

## Data Availability

The data that support the findings of this study will be available from the corresponding author, [LW], upon reasonable request.

## Abbreviations

BIS: Body Image Scale
Fex-Can: Fertility and Sexuality following Cancer
Fex-Can Cohort: Fex-Can Population-based Cohort study
GDPR: General Data Protection Regulation
HADS: Hospital Anxiety and Depression Scale
PROMIS: Patient-Reported Outcomes Measurement Information System®
RCAC: Reproductive Concerns After Cancer scale
RCT: Randomized Controlled Trial
SPAR: The Swedish Population Register
SexFS: Sexual Function and Satisfaction measure
